# Hospitalisation by tick-borne diseases in the last 10 years in two hospitals in South Spain: analysis of tick exposure data collected in the Emergency Department

**DOI:** 10.1017/S095026881900147X

**Published:** 2019-08-23

**Authors:** M. Rivera-Izquierdo, L. M. Martín-delosReyes, A. J. Láinez-Ramos-Bossini, P. Ruiz-Díaz, E Casado-Fernández, A. Bueno-Cavanillas, V. Martínez-Ruiz

**Affiliations:** 1 Service of Preventive Medicine, Hospital Clínico San Cecilio, Granada; 2 Department of Preventive Medicine and Public Health, University of Granada, Spain; 3 Department of Radiology, Hospital Virgen de las Nieves, Granada; 4 Service of Documentation, Hospital Clínico San Cecilio, Granada; 5 Instituto de Investigación Biosanitaria de Granada (ibs.GRANADA), Spain; 6 CIBER de Epidemiología y Salud Pública (CIBERESP), Spain

**Keywords:** Emergency Department, epidemiology, ticks, tick-borne diseases

## Abstract

Tick-borne diseases (TBDs) can sometimes cause severe symptoms and lead to hospitalisation, but they often go unnoticed in the Emergency Department (ED). The aim of this study was twofold: (i) to describe the profile of patients hospitalised by TBDs; and (ii) to evaluate the data collected in the medical records from the ED in order to analyse their potential clinical consequences. A total of 84 cases that included all TBD diagnoses registered in the ED records were identified and analysed. These corresponded to all the hospitalisations by TBDs in the last 10 years (2009–2019) in two tertiary hospitals in Granada, Spain. Statistical analyses were made using RStudio. Coinciding with the absence of patient's report of exposure to ticks, 64.3% of TBDs were not suspected in the ED. Intensive care unit admission was required in 8.3% of cases, and the mortality rate was 2.4%. Non-suspected cases showed longer hospital stay (*P* < 0.001), treatment duration (*P* = 0.02) and delay in the initiation of antibiotic treatment (*P* < 0.001). Our findings indicate that symptoms associated with TBDs are highly non-specific. In the absence of explicit information related to potential tick exposure, TBDs are not initially suspected. As a consequence, elective treatment administration is delayed and hospitalisation time is prolonged. In conclusion, our results highlight the importance of addressing potential exposure to ticks during the ED contact with patients presenting with febrile syndrome.

## Introduction

Tick-borne diseases (TBDs) often represent a challenge for emergency clinicians [1]. Numerous studies have reported an underdiagnosis of these diseases worldwide [1–4]. Because the presentation of TBDs is generally non-specific, they can often be misdiagnosed, especially in the early stages of the illness. Besides, the majority of patients affected by tick bites do not develop any TBD in the end. Since tick bites are usually painless, patients can be unaware of them or confuse the local reaction with the bite of another arthropod [5]. Moreover, tick bites remain undetected due to the small size of the parasite in some stages (i.e. nymphs) and the location of bites in hidden body areas (i.e. head/hair). In addition to these reasons, TBDs can be overlooked in the Emergency Department (ED) because risk and exposure are perceived as minimal (e.g. having been in the countryside or having pets at home) [1]. However, many TBDs can be life-threatening and have long-lasting effects if not treated on time [6, 7].

Although Primary Care is the first level of health care in the Spanish National Health System, most patients with a serious disease visit the ED due to its free access, either voluntarily or derived from a health centre. For this reason, ED clinicians have an essential role in the identification and early treatment of severe TBDs.

In Southern Europe, particularly in Spain, most TBDs correspond to rickettsioses (incidence 0.36/1 00 000 individuals) [8] and, in Mediterranean areas, *Rickettsia conorii* causes the Mediterranean spotted fever (MSF). The clinical manifestations of rickettsioses range from self-limiting symptoms to fatal diseases [9]. The number of bites by the vector that transmits MSF, *Rhipicephalus sanguineus*, is higher during the warmer months of the year and, in the last decades, an increase in the number of fatal cases has been reported [10]. Early diagnosis and treatment of TBDs have proved to reduce their morbidity (in terms of duration of the disease and antibiotic treatment) and mortality [11]. Nevertheless, differentiating TBDs, especially rickettsial infections, from other febrile syndromes can be very difficult if only clinical data are considered. Moreover, standard diagnostic tests have a low sensitivity, particularly in the early stages of the illness [12]. The diagnosis of TBDs is easier in the presence of an inoculation eschar or *tache noire*, a painful necrotic lesion that typically appears at the tick bite site. Nonetheless, these lesions do not appear in all cases of severe disease [13]. Apart from *R. conorii*, a growing increase in other tick-borne *Rickettsia* species has been reported in Europe [14]. Environmental and climatic changes, among other factors, may contribute to the emergence and spread of these other rickettsioses, which justifies the need for raising suspicion of TBDs [15]. Moreover, some studies have reported an increase in the time periods in which the diagnosis of TBDs is more frequent [14–16]. The recent cases of Crimean–Congo haemorrhagic fever (CCHF) virus in Spain are a clear example of it [16]. In fact, bird migration due to climatic changes has been pointed out as the main cause of the CCHF spread from African countries to Spain [16]. Clinically, CCHF is characterised by fever, coagulopathy and hepatitis. Haemorrhagic symptoms and long-lasting fever should raise suspicion of TBD.

The next most frequent TBD in Spain (incidence 0.25/1 00 000 individuals) is Lyme disease (LD), which is caused by *Borrelia burgdorferi* and is generally transmitted by the vector *Ixodes* spp. LD is also highly variable in terms of symptoms and severity, and represents the most frequent TBD in the USA [17].

In many cases, the underdiagnosis of TBDs results from the absence of epidemiological background on possible contact with ticks collected by the ED clinicians in non-specific febrile syndromes [4]. Since the risk of long-term morbidity and mortality increases with delayed treatment, some authors have emphasised the need for keeping a high index of suspicion for TBDs, establishing a presumptive diagnosis without waiting for confirmatory test results, and promptly starting treatment with doxycycline [4]. Particularly during the warmer months of the year, a detailed history addressing recent activities and travel, and a thorough physical examination would help narrow the diagnosis [1].

The hypothesis of the present study was that the absence of data related to tick exposure in the ED medical records leads to a delay in the diagnosis and has a negative impact on the eventual treatment of TBDs, increasing the time of hospitalisation as well as morbidity and mortality. The aim of this study was twofold. On the one hand, it aimed to collect and describe all cases of hospitalised TBD in two tertiary hospitals in a city of Southern Spain (Granada) in the last 10 years, based on feasibility criteria, and observe the collection of epidemiological information related to tick exposure in EDs. On the other hand, it aimed to analyse its impact in terms of time of hospitalisation and treatment delay, and identify possible factors to improve the medical assistance of patients with TBDs.

## Methods

### Study design and data collection

**Fig. 1.**
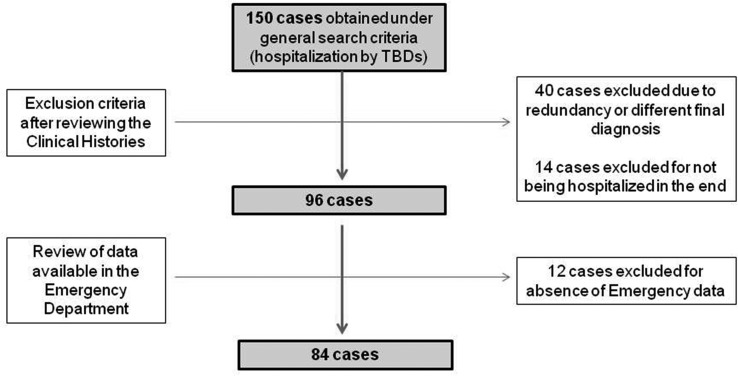
Flow diagram followed in the study.

The study designed was a hospital case series. Data were collected by a professional search performed by the Service of Documentation at the Hospital Clínico San Cecilio, in Granada, Spain. Clinical histories were collected and anonymised. The inclusion criteria were: having been hospitalised, having data available from the Emergency Report and having a final diagnosis of TBD (only LD and MSF cases were detected). To gather the initial data, the following parameters were used: ‘tick’ or ‘Lyme disease’ or ‘Mediterranean spotted fever’ or ‘Crimean-Congo’ or ‘rickettsiosis’ or ‘rickettsia’ or ‘borreliosis’ or ‘borrelia’ in two different databases: one from the ED and the other from hospitalisations, including all cases from both hospitals from 1 January 2009 to 1 January 2019. Initially, 150 cases were found in the two databases. After discarding duplicates and conducting a comprehensive review of the clinical histories, 96 cases of hospitalisation by different TBDs were detected. No recorded data from the ED were found in 12 more patients. Thus, a total of 84 patients were eventually included in the study. LD and MSF had a specific medical code according to the *International Classification of Diseases, Tenth Revision* (ICD-10). The flow chart of data collection can be observed in Figure 1.

Next, the selected medical records were mined to collect demographic information, data related to the symptomatology of the patients, the information collected in the ED and their evolution during hospitalisation.

### Descriptive analysis

Once the database was obtained and refined, statistical analyses were carried out using RStudio free software through its application RCommander, version 3.4.2.

First, a descriptive analysis of socio-demographic variables was performed, including age, sex, year and month of the episode and name of the admitting hospital. Likewise, the variables related to the emergency care of the episode were analysed, including patient's report of bite or contact with ticks, bite or ticks identification in the examination, area of the body where the bite was found and frequency distribution of the symptoms associated with the process in the ED. Similarly, other variables were registered in the database, including final diagnosis (MSF, LD or others), serological confirmation, unit and time of hospitalisation, need of intensive care and time until the initiation of elective treatment. Finally, epidemiological data reported in the ED were collected, namely activity considered at risk for contact with ticks, and suspicion of TBDs. To evaluate and collect this suspicion, the presence of data asked the patient and collected in the ED report was considered.

### Analysis of the main variable

In order to evaluate the differences in the hospitalisation process regarding the epidemiological information collected in the ED clinical history, the variables average time of hospitalisation and average time of delay in the start of antibiotic treatment were compared with the presence/absence of information related to possible contact with ticks (suspected cases *vs.* non-suspected cases). The suspicion was analysed attending to two variables: the presence (yes/no) of information given by the patient, and the presence (yes/no) of TBDs suspected by the clinicians in the ED report. The presence of the word ‘tick’ in the ED report, or serological analyses related to TBDs were included as ‘suspected cases’. To analyse the differences, a comparison test of two means was used (*t* tests) after confirmation of application conditions. Normality and homoscedasticity were confirmed by Kolmogorov–Smirnov and Levene tests. Also, *χ*^2^ tests were applied to analyse the relationship between suspicion of TBD, bite report by the patient and symptoms. Finally, Pearson's correlation test was used to compare quantitative variables (age, date of the episode, treatment duration, time between ED contact and the initiation of antibiotic treatment and total days of hospitalisation).

## Results

### Descriptive analysis

The analysis of socio-demographic variables (sex, age, month of the episode and admitting hospital) and general information collected in the ED (report contact with ticks, clinician's suspicion at the ED, risk activity and associated symptomatology) are shown in Table 1. The mean age was 53.74 years (s.d. = 20.1; min = 3, max = 90). In addition, age was not homogeneously distributed, with 59.5% of patients aged between 40 and 80. Age and sex correspond to the data offered by the *Basic Minimum Data Set* of other Spanish hospitals [18], regarding patients hospitalised by TBDs. Likewise, an average of 5.23 days (standard deviation 3.21) was observed between the bite or initial symptomatology and the patient's contact with the health services. All patients (100%) initially went to the ED (i.e. not other health services such as Primary Care). No report of tick bite or contact with ticks (non-suspected cases) was identified in 64.3% of the Emergency Reports.

**Table 1.** Description of the variables collected in the Emergency Department

Variables	Frequency		Percentage	
Sex				
Female	36		42.86%	
Male	48		57.14%	
Month of arrival at the ED				
January–March	4		4.76%	
April–June	24		28.57%	
July–September	46		54.76%	
October–December	10		11.90%	
Hospital^a^				
HCSC	34		40.48%	
HUVN	38		45.24%	
Both*	12		14.29%	
Most frequent anatomical areas where tick bites were observed				
Head and neck	10		13.5%	
Shoulders	8		10.8%	
Back	8		10.8%	
Chest and abdomen	0		0%	
Armpits and upper extremities	6		8.1%	
Groins and hips	12		16.2%	
Lower extremities	14		18.9%	
Report of tick bite or contact with ticks by the patient				
Yes	30		35.7%	
No	54		64.3%	
The tick-borne disease was suspected on ED first contact				
Yes	30		35.7%	
No	54		64.3%	
Risk activity associated with the tick bite				
No information collected	36		42.86%	
Picnic/trip in the countryside	24		28.57%	
Have dogs with ticks	18		21.43%	
Contact with wild animals	6		7.14%	
Associated symptomatology				
Fever	76		90.48%	
Exanthema	74		88.10%	
Neurological/behavioural symptoms	44		52.38%	
Arthralgia/myalgia	32		38.10%	
Dyspnoea	22		26.19%	
Abdominal pain	14		16.67%	
Variable	Mean	Minimum	Maximum	Standard deviation
Age	53.74	3	90	20.1

Data of all the cases (84) are presented as *n* (%) for categorical variables and mean (minimum, maximum, standard deviation) for continuous variables.

^a^HSCS and HUVN correspond to the included hospitals of Granada: Hospital Clinico San Cecilio and Hospital Universitario Virgen de las Nieves. Both* corresponds to the period of 2015 in which, due to a political project, both hospitals were merged during a year. In 2016, the project was cancelled and the hospitals returned to their original independence.

The most common anatomical areas where bites were observed are also presented in Table 1. Head and neck, shoulders and groins and lower extremities gathered the largest number of bites. All identified TBDs corresponded to bacterial diseases. The most frequent clinical diagnosis was MSF (88% of the cases), 44% of which had serological confirmation afterwards, followed by LD (4.76%). No specific diagnosis was provided in the remaining cases (7.14%), which were identified as *fever due to tick bite*. The most frequent hospitalisation admission services were Infectious Diseases Service (40.48% of the cases) and Internal Medicine Service (33.33%). The remaining 26.19% of the cases were admitted to other services based on the symptoms observed.

The mean hospitalisation time was 8.36 days (s.d. = 4.74) and the delay time at the start of antibiotic treatment was 1.45 days (s.d. = 0.63). In total, 17.07% of the patients needed intensive care due to shock (71%) or hypotension (29%). Of the total 84 cases, two resulted in death and one in abortion. The rest of the patients were discharged after hospitalisation to continue the antibiotic treatment at home.

### Analysis of the data related to tick exposure

The main variable of this study was the presence or absence of information related to contact with ticks in the ED report, which was classified as a suspected case (SC) or non-suspected case (NSC). To validate parametric tests, the average hospitalisation time (*D* = 0.18, *P* = 0.01) and the mean delaying days of antibiotic treatment (*D* = 0.38, *P* < 0.01) were analysed by Kolmogorov–Smirnov and Levene tests, which indicated normality and homoscedasticity of these variables.

To compare the mean time of hospitalisation depending on the suspicion, *t* tests were applied (Table 2). Statistically significant differences were observed with longer stays in the NSC group (x̄_NSC_ = 10.1 *vs.* x̄_SC_ = 5.2 days, *P* < 0.001).

**Table 2.** Differences detected between suspected and non-suspected cases at the first contact with the Emergency Department (ED)

Variable	SC	NSC	*P*-value
Days of hospitalisation	5.2 (1.22)	10.1 (5.31)	<0.001^a^
Days between ED contact and antibiotic treatment initiation	1.1 (0.3)	1.8 (0.76)	<0.001^a^
Days of treatment (including hospital and home treatment)	11.8 (4.3)	15.1 (6.2)	0.02^a^
Cases requiring treatment in the intensive care unit	2 (7)	5 (12)	0.6^b^
Presence of fever	30 (100)	44 (84)	0.23^c^

Data are presented as mean (standard deviation) for continuous variables and *n* (%) for categorical variables.

Legends for illustrations.

^a^Comparison was determined by *t* test for continuous variables after checking the application conditions. Normality and homoscedasticity were assessed by Kolmogorov–Smirnov and Levene tests.

^b^Comparison was determined by Fisher's exact test for categorical variables with <80% of expected values >5.

^c^Comparison was determined by *χ*^2^ test after validating the application conditions.

Similarly, significant differences were found in the time to start the antibiotic treatment. On average, the time to start the elective treatment in NSCs was 0.7 days longer than in SCs (*P* < 0.001). The total days of treatment, including both hospital and domiciliary treatment, yielded 3.3 more days on average in NSCs (*P* = 0.02). No significant differences were observed in the total number of cases or in the degree of clinical suspicion over the 10 years of study.

On the other hand, Pearson's correlation was used to analyse the relationship between the delaying time of antibiotic treatment and the average hospitalisation time. A positive relationship was found (*r* = 0.43, *P* < 0.01).

Finally, *χ*^2^ test showed a clear relationship between the suspicion at the ED and the report of bite by the patient (*P* < 0.01), with an absolute match in the contingency table. No significant differences in the symptoms were found. In this study, no case presented haemorrhagic symptoms or signs described as clinical criteria of CCHF.

## Discussion

Sex, age and frequency of the diagnosis of this study agree the general data for TBDs collected through *Basic Minimum Data Sets* of other Spanish hospitals [18]. Similarly, the symptoms associated with TBDs coincide with those reflected in other studies [1–4]. In summary, the symptomatology described is highly unspecific, with fever as the most frequent symptom.

Our results suggest that suspicion of TBD at the initial contact with the ED is associated with a decrease in the time of hospitalisation, the total time of treatment and the time of delay until the administration of antibiotics. In this vein, lower sanitary costs and higher quality of care for these patients could be achieved if more attention was paid to symptoms potentially linked with TBDs.

Likewise, our results showed a higher percentage of patients needing intensive care in the group of NSC (12% *vs.* 7%). However, no statistical differences were found, probably due to the low proportion of patients that required this type of medical attention (only seven out of 84).

Among the limitations of the study, it is worth highlighting the sample size, which corresponds to the total number of patients hospitalized by TBDs in the two tertiary hospitals in Granada in the last 10 years. This reduces the applicability of our results to other European and world regions where TBDs have a different prevalence, aetiology or distribution. Only severe cases were considered for this study, i.e. patients that ended up being hospitalised-, because these provided more detailed information. Similarly, the hospitals and the interval of study were chosen according to feasibility criteria. The databases from the Documentation Service had no reliable coded data before 2009. Only cases detected in the ED were included, i.e. cases of TBD from Primary Care or underdiagnosed cases were not considered in this study. The source of data (i.e. medical records) was another limitation for the collection of detailed information. Only 44% of clinical diagnoses were confirmed by serology, or either this information was not registered electronically. Also, 12 out of 96 (12.5%) cases were missing due to the absence of data in the medical records, which may represent a bias due to missing information.

It is important to mention that all the SCs correspond to patients that actively reported contact with ticks. This implies that, in the absence of information provided by patients, clinicians tend to ignore the possibility of contact with ticks.

Considering the temporal distribution of TBDs observed, the low percentage of clinicians that explore potential contact with ticks, and the lack of specificity of the symptoms, our results indicate that ED clinicians have a relevant role in the approach of TBDs. In this vein, when a patient presents with non-specific fever, especially during the warmer months of the year, asking about possible contact with ticks could lead to suspecting this disease from the very beginning. According to our findings, this early suspicion could result in shorter hospitalisation time and earlier start of antibiotic treatment, which shall lead to better prognosis, fewer side effects and lower healthcare costs. However, provided that this study is a case series, we can only propose these hypotheses and highlight the relationships observed, but analytic observational studies (e.g. cohort or case–control studies) should be conducted to confirm causality. In the same way, local interventions to keep ED clinicians up to date on the most frequent TBDs in their region after evaluating their competencies through interviews or questionnaires (e.g. what measures they would take if a patient presented with TBD symptoms) could have a positive impact on the management of patients with TBDs.

Finally, after the alarm generated in Spain due to recent cases of CCHF for the presence of a new vector [16], it was proposed to use this study to analyse the presence of haemorrhagic symptoms in the most frequent TBDs. The data showed that none of the 84 patients presented haemorrhagic symptoms or compatible analytic findings such as a decrease in the number of platelets. On the basis of our findings, we suggest that the presence of tick bite and haemorrhagic symptoms combined justifies the activation of an alarm suspecting CCHF. In any case, the execution of sanitary training courses promoted by Emergency Services or Preventive Medicine Services may play a crucial role in updating and improving the early suspicion of TBDs, which could result in economic savings in health and better health care.

In conclusion, the symptoms associated with TBDs are highly non-specific. In the absence of explicit epidemiological information related to exposure to ticks, TBDs are not initially suspected in the ED. This is related to a delay in the start of elective antibiotic treatment and to an increase in the hospitalisation time. The results of this study reinforce the idea that the clinical history of a patient with febrile syndrome in the ED should include questions addressing potential contact with ticks. Also, our results indicate that haemorrhagic symptoms are very unlikely in the most common TBDs, which calls for alarm activation to rule out CCHF if haemorrhagic signs or symptoms are present.
